# Book Review

**DOI:** 10.1155/S1463924680000661

**Published:** 1980


**Annual Review of Clinical Biochemistry Volume 1**
*Edited by David M. Goldberg. John Wiley & Sons, 1980, Chichester.*
*pp 379, £12, ISBN 0471-040-363*

The first chapter is concerned with quality control, laboratory management and reference values. Its author, P. M. G. Broughton, has managed by careful selection of his sources to cover most of the important papers published in 1978 covering this subject and at the same time presenting a readable review in its own right which will inform the reader as well as act as a pointer to the literature. The objectives of an efficient clinical chemistry laboratory include the production of data for clinical use which are accurate, can be repeated precisely, are relevant to the problem, are timely in relation to clinical decisions, are interpretable and are not wasteful of resources. Anyone reading this chapter will find something of interest under all these headings and referring to the original references will find more detailed and, usually, authoritative discussion. Emphasis has been placed on problems associated with the use of commercial quality control material, the impact of reference methodology on the interpretation of consensus values in external quality assurance schemes and the influence of subpopulation and other variables in the estimates of 'normal' ranges.

Harry Pardue and Stanley Deming have contributed the chapter on instrumentation and computing. They have reviewed much of the interesting development in analytical chemistry applied to medicine including multilayer-film systems, flow-injection systems without air segmentation, RIA using magnetised particle separation, electrochemical detection systems including imaging detectors (to which Professor Pardue has made significant contributions himself). These exciting analytical systems together with microprocessor control are likely to bring a new generation of instruments to clinical laboratories. In addition the authors have chosen to limit their review of computer studies to distributed laboratory computing glucose controlled insulin infusion, optimisation studies, choice of sequential tests, and the application of computers to GC-MS, all of which are interesting topics. They have also brought to the attention of their readers the recent critical reviews of the use of regression data in the comparison of analytical methods. Whilst any worker in a particular field will be aware of most of the references quoted there will be much in less familiar fields which will stimulate and inform. The other chapters, which are of equal value, are concerned with advances in the application of biochemistry to clinical medicine.

*M. G. Rinsler*

### Japanese Journal of Clinical Laboratory Automation

### In volume 5, number 3, this journal will contain the following items:

**Original Papers**

1. Evaluation Studies on Hitachi 726 Automatic Analyser
Mitsuo Sekiguchi, *et al.* (2)
2. New Colorimetric Determination of Transaminase Activity by Use of Pyruvate Oxidase and its Application to the Automated Analyser
Shozo Tabata, *et al.* (5)
3. A New Rapid Determination Method for CPK-MB with the Du Pont Automated Clinical Analyser (aca)
Tetsu Tsuzi, *et al.* (8)
4. Chloride Meter Applied to Auto Chemist
Kenzo Fujiwara, *et al.* (11)
5. Instrumentation for Continuous Potentiometric Measurement of Potassium Concentration and pH in Whole Blood During Open Heart Surgery
Masayuki Totani, *et al.* (14)
6. Some Problems on the Determination if Ionised Calcium with "NOVA-2" by ion Selective Electrode
Yoshiko Kurosake, *et al.* (17)
7. Enzymatic Method for the Determination of Inorganic Phosphate: Application for ABBOTT-VP
Tatsuo Hara, *et al.* (20)
8. Determination of Iron and Total Iron Binding Capacity in Serum by Ferro Chem (Model 3050)
Miyoko Saito, *et al.* (23)
9. The Evaluation of Monitor KDA
Takehisa Nagato, *et al.* (28)
10. Evaluation of Microlyser - an Automatic Analyser of the Micro Clinical Chemistry
Tsuyoshi Sasamura, *et al.* (32)
11. Some Problems on RIA Total System-ALOKA
Toshiaki Nakai, *et al.* (35)
12. Evaluation of Homogenous Enzyme Immunoassay of Serum-Thryoxine with Rotochem II a 36 Analyser
Akira Abe, *et al.* (38)
13. Determination of Steroids in Urine-Hydrolysis of Steroid Glucuronides with Use of a β-Glucuronidase Tube Reactor
Akira Kanai, *et al.* (41)
14. A New Apparatus "PSP-AUTO™" for the Phenolsulfonphthalein Excretion Test
Junko Nakamoto, *et al.* (44)
15. Evaluation of Urine Analyser "Clinilab", Comparison with "Hitachi-678" Urinalyzer
Yoshiaki Iizuka, *et al.* (48)
16. Evaluation of the Automatic Identification of Enterobacteriaceae by a New Apparatus "BACOMPTE-E"
Tsuneko Ohata, *et al.* (51)
17. The Application of Bactobridge to Rapid Assay for Antibiotics in Serum
Hidehiro Tsuneoka, *et al.* (54)
18. Enzymatic Analysis of Free Fatty Acid by the Rotochem II a Centrifugal Fast Analyser
Akira Abe, *et al.* (58)
19. A New Automatic Glucose Analyser Using a Micro Hematocrit Tube
Kiyoko Ikeda, *et al.* (61)

